# Centrin 2: A Novel Marker of Mature and Neoplastic Human Astrocytes

**DOI:** 10.3389/fncel.2022.858347

**Published:** 2022-04-29

**Authors:** Elisa Degl’Innocenti, Tino Emanuele Poloni, Valentina Medici, Luca Recupero, Claudia Dell’Amico, Eleonora Vannini, Ugo Borello, Chiara Maria Mazzanti, Marco Onorati, Maria Teresa Dell’Anno

**Affiliations:** ^1^Fondazione Pisana per la Scienza ONLUS, San Giuliano Terme, Italy; ^2^Department of Translational Research and of New Technologies in Medicine and Surgery, University of Pisa, Pisa, Italy; ^3^Department of Neurology and Neuropathology, Golgi-Cenci Foundation and ASP Golgi-Redaelli, Abbiategrasso, Italy; ^4^Unit of Cell and Developmental Biology, Department of Biology, University of Pisa, Pisa, Italy; ^5^CNR Institute of Neuroscience, Pisa, Italy

**Keywords:** neural stem cells, centrin-2, centrosome, astrocytes, glioblastoma multiforme

## Abstract

As microtubule-organizing centers (MTOCs), centrosomes play a pivotal role in cell division, neurodevelopment and neuronal maturation. Among centrosomal proteins, centrin-2 (CETN2) also contributes to DNA repair mechanisms which are fundamental to prevent genomic instability during neural stem cell pool expansion. Nevertheless, the expression profile of CETN2 in human neural stem cells and their progeny is currently unknown. To address this question, we interrogated a platform of human neuroepithelial stem (NES) cells derived from *post mortem* developing brain or established from pluripotent cells and demonstrated that while CETN2 retains its centrosomal location in proliferating NES cells, its expression pattern changes upon differentiation. In particular, we found that CETN2 is selectively expressed in mature astrocytes with a broad cytoplasmic distribution. We then extended our findings on human autoptic nervous tissue samples. We investigated CETN2 distribution in diverse anatomical areas along the rostro-caudal neuraxis and pointed out a peculiar topography of CETN2-labeled astrocytes in humans which was not appreciable in murine tissues, where CETN2 was mostly confined to ependymal cells. As a prototypical condition with glial overproliferation, we also explored CETN2 expression in glioblastoma multiforme (GBM), reporting a focal concentration of CETN2 in neoplastic astrocytes. This study expands CETN2 localization beyond centrosomes and reveals a unique expression pattern that makes it eligible as a novel astrocytic molecular marker, thus opening new roads to glial biology and human neural conditions.

## Introduction

Neural stem cells, including neuroepithelial and radial glial cells, are by definition multipotent, and their proliferation during intrauterine life is critical for the formation of a pool of progenitors committed to differentiate into mature neurons and glial cells (Gotz and Huttner, 2005). Disruption of mitosis events during neural stem cell expansion, due to either environmental or genetic insults, provokes inevitable alterations in neurodevelopment which lead to severe congenital malformations (Faheem et al., 2015; Onorati et al., 2016; Baggiani et al., 2020).

As microtubule nucleation centers, centrosomes not only orchestrate mitotic spindle assembly, thus ensuring the correct partition of genetic material in proliferating neural stem cells, but also play a pivotal role in the development of brain cytoarchitecture, mediating neuronal migration and layering, and ensuring the cytoskeletal structuring and polarization of newly born neurons (Higginbotham and Gleeson, 2007; Kuijpers and Hoogenraad, 2011). Given these elements, it is not surprising that several neurodevelopmental disorders, including microcephaly and lissencephaly, can be ascribed to mutations or deletions in centrosomal genes (Kuijpers and Hoogenraad, 2011).

Among several centrosomal proteins, centrins (also known as caltractins) are small calcium-binding proteins that have attracted much attention for their involvement in multiple basic cellular functions, such as cell duplication (Salisbury et al., 2002), ciliogenesis, and DNA repair (Khouj et al., 2019). Among the four isoforms described in mammals (CETN1-4), centrin-2 (CETN2) is specifically known to participate in nucleotide excision repair (NER) mechanisms as part of the *Xeroderma pigmentosum* C (XPC) complex and, accordingly, *CETN2* deletions result in inefficient DNA damage resolution and repair (Dantas et al., 2012). Recurrent DNA damage in neural stem cells has been reported to trigger neuronal degeneration or cancer (Alt et al., 2017); therefore, the proper activation of DNA repair machinery is fundamental for healthy neurodevelopment and disease prevention. Although CETN2 has largely been studied in lower eukaryotes (Salisbury, 2007), descriptions of CETN2 in the central nervous system (CNS) have been so far limited to ciliated cells, including photoreceptors. Specifically, it has been reported that *Cetn2* gene deletion leads to dysosmia, due to impaired olfactory cilia trafficking, or hydrocephalus, caused by disrupted ependymal cilia planar polarity (Ying et al., 2014). Similarly, *Cetn2* knock-out has been described to destabilize the photoreceptor axoneme, leading to complete retinal degeneration (Ying et al., 2019).

Despite its role in cell division and DNA repair mechanisms, the expression profile of CETN2 in human neural stem cell populations has never been investigated so far. Here, in order to address this point, we took advantage of a unique platform of human neuroepithelial stem (NES) cells derived from *post mortem* developing human brain. NES cells can be easily propagated *in vitro* preserving unaltered multipotency and retaining the transcriptional profile of their *in vivo* counterpart (Conti and Cattaneo, 2010; Onorati et al., 2016; Dell’Anno et al., 2018).

CETN2 exhibited a focal centriolar expression in NES cells; however, its subcellular localization in NES cell-derived neuronal and glial progeny provided unexpected results. In particular, we pointed out a diffuse cytoplasmic distribution of CETN2 in mature astrocytes with a pattern very similar to glial fibrillary acidic protein (GFAP), and the same results were also recapitulated in astrocytes derived from pluripotent sources. Based on these findings, we also extended our investigation to healthy human brain sections and to pathological conditions with a large abundance in astrocytes, finding in glioblastoma multiforme (GBM) a prototypical disease. We found that, while CETN2 was not expressed ubiquitously in all astrocytes from control brains, it exhibited a focal concentration in malignant brain cancer. Although others have previously described the presence of CETN2 in multiple subcellular compartments outside centrioles, including cytosol and nucleus (Paoletti et al., 1996), the specific expression of CETN2 in human astrocytes has never been reported.

Therefore, this study demonstrates for the first time, to the best of our knowledge, that CETN2 is expressed in human CNS with an anatomical localization pattern that exceeds ependymal cells and photoreceptors and unravels its specificity as an astrocyte molecular marker.

## Materials and Methods

### Human Fetal Tissue Procurement

Human-induced pluripotent stem (iPS) cell line was established from skin fibroblasts, as already reported by Sousa et al. (2017). NES cells were derived from prenatal cortical tissue, as already reported by Onorati et al. (2016). Fetal material for the collection of NES cells and fibroblasts was obtained from the Joint MRC/Wellcome Trust Human Developmental Biology Resource upon appropriate informed consent. Tissue procurement and handling were performed according to the NIH guidelines for the acquisition and distribution of human material for biomedical research purposes with the prior approval of Human Investigation Committees and Institutional Ethics Committees from which samples were obtained (University of Pisa Review No. 29/2020). All available non-identifying information was recorded, and the tissue was handled in accordance with the ethical guidelines and regulations for the research use of human brain tissue set forth by NIH and the WMA Declaration of Helsinki.

### Maintenance and Differentiation of Human Neuroepithelial Stem Cell Lines

The NES cells utilized in this study were cultured, as previously reported by Onorati et al. (2016) and Dell’Anno et al. (2018). Briefly, NES cells were kept in proliferation in T25 flasks coated with poly-L-ornithine (0.01%, Sigma, #P4957), laminin (5 μg/ml, Thermo Fisher Scientific, #23017-015), and fibronectin (1 μg/ml, Corning, #354008). NES medium was composed as follows: Dulbecco’s modified Eagle’s medium (DMEM)/F12 (Thermo Fisher Scientific, #11330-032) with the addition of B27 supplement (1:1,000, Thermo Fisher Scientific, #17504044), N2 supplement (1:100, Thermo Fisher Scientific, #17502-048), 20 ng/ml fibroblast growth factor 2 (FGF2) (Thermo Fisher Scientific, #13256029), 20 ng/ml epidermal growth factor (EGF) (Thermo Fisher Scientific, #PHG0311), 1.6 mg/ml glucose, 20 μg/ml insulin (Sigma, #I9278), and 5 ng/ml brain-derived neurotrophic factor (BDNF) (Thermo Fisher Scientific, #PHC7074). When cells were expanded, 10 μM Rock Inhibitor (Y27362, Sigma, #SCM075) was added to the NES medium to increase cell viability. Cells were passaged in the ratio of 1:2 for about 1 or 2 times a week using 0.25% trypsin (Thermo Fisher Scientific, #25200056) inactivated using 10% fetal bovine serum (FBS)/phosphate-buffered saline (PBS) solution. Half volume medium was changed every 2–3 days to allow medium conditioning.

Neuronal differentiation of NES cells was performed in two steps. For the pre-differentiation step, NES cells were seeded at a density of 0.5 × 10^5^ cells/cm^2^ in a T25-coated flask in NES medium without EGF and FGF2. After 5–7 days, cells were dissociated and replated at a density of 0.8 × 10^5^ cells/cm^2^ onto Lumox plates (Sarstedt, #946077331) in Neurobasal medium (Thermo Fisher Scientific, #21103049) combined with 1:1 DMEM/F12 medium (Thermo Fisher Scientific, #11330-032). The differentiation medium was enriched with B27 (1:100), N2 (1:200), and BDNF (20 ng/ml). The half volume of neuronal medium was changed every 2–3 days, and neurons were differentiated up to 80 days. Both NES medium and differentiation medium were supplemented with antibiotics (Penicillin-Streptomycin 1:100, Thermo Fisher Scientific, #15070063).

### Derivation and Maintenance of Induced Pluripotent Stem Cell Lines

The iPS cells, generated as previously described through episomal reprogramming (Sousa et al., 2017), were maintained in StemFlex Basal medium (Thermo Fisher Scientific, #A3349201) or Essential 8™ medium (Thermo Fisher Scientific, #A2858501) on Matrigel-coated culture plates (1:60, Corning, #356234). To preserve pluripotency properties, iPS cells were passaged every 5–6 days at a 1:6–1:8 ratio. To induce colonies detachment, cells were incubated at room temperature (RT) with EDTA (0.5 mM) for 3–5 min. After EDTA removal, culture vessels were rinsed with cell media to collect small clumps of cells. Finally, iPS cells were plated according to the optimal split ratio in a new Matrigel-coated culture plate.

### Neural Induction and Differentiation

The iPS cells were driven toward cortical fate through the application of the dual small mothers against decapentaplegic (SMAD) inhibition protocol (Chambers et al., 2009; Sousa et al., 2017). As previously described, iPS cells were dissociated into a single cell-suspension with pre-warmed Accutase (Corning, #25-058-CI) and plated onto Matrigel-coated dishes (1:50, Corning, #356231) at the density of 2 × 10^5^ cells/cm^2^ in StemFlex basal medium (Thermo Fisher Scientific, #A3349201) supplemented with 10 μM Y-27632 (STEMCELL Technologies, #72308). Once 90–95% confluent, cell medium was replaced with Neural Induction medium [mixture 1:1 DMEM/F-12 (Thermo Fisher Scientific, #31330095) and Neurobasal medium (Thermo Fisher Scientific, #21103049)] supplemented with N-2 (1:100), B-27 (1:50), 20 μg/ml insulin, L-glutamine (1:100, Thermo Fisher Scientific, #25030-081), MEM non-essential amino acids (1:100, Thermo Fisher Scientific, #11140-050), and 2-mercaptoethanol (1:1,000, Thermo Fisher Scientific, #31350010). 100 nM of LDN-193189 (STEMCELL Technologies, #72144), 10 μM of SB-431542 (Merck, #616464-5MG), and 2 μM of XAV939 (STEMCELL Technologies, #72674) were added to efficiently induce cerebrocortical fate. The neural induction medium was replaced daily until day 11. On day 12, cells were detached with Accutase and replated in drops onto poly-D-lysine (Sigma, #P6407, 10 μg/ml) and laminin (Invitrogen, #23017-015, 3 μg/ml)-coated chamber slides (Sarstedt, #94.6140802) at the density of 1 × 10^5^ cells/drop. Neural induction medium has been substituted with terminal differentiation medium containing Neurobasal medium supplemented with N-2 (1:100), B-27 (1:50), L-glutamine (1:100), BDNF, and Y-27632 (10 μM) to increase cell viability. Cell medium was then partially refreshed every 3–4 days until terminal differentiation (day 80).

### GL261 Cell Culture

GL261 cells were grown, as previously reported by Vannini et al. (2021). Briefly, GL261 cells were expanded in complete DMEM (Gibco, #12634-010) containing 10% newborn calf serum (Gibco, #26010066), 4.5 g/L glucose (Thermo Fisher Scientific, #A2494001), 2 mM glutamine (Thermo Fisher Scientific, #25030149), 100 IU/ml penicillin, and 100 mg/ml streptomycin (Thermo Fisher Scientific, #10378016) at 37°C in 5% CO_2_ with media changes three times per week.

### Immunofluorescence

The NES cells, iPS cells, and their neuronal and glial progeny were washed with PBS and fixed with 4% formaldehyde for 10 min at RT. After three additional washes in PBS, cells were left in blocking solution (PBS supplemented with 1% horse serum and 0.1% Triton) for 1 h at RT. Cells were incubated with primary antibodies ON at 4°C. Primary antibodies were diluted as follows: Nestin (1:200, R&D systems, #MAB1259), SOX1 (1:200, Cell Signaling, #4149), SOX2 (1:400, Merck-Millipore, #AB5603), MKI67 (1:300, Abcam, #16667), pericentrin (PCNT) (1:1,000, Abcam, #ab28144), CETN2 (1:500, Merck-Millipore, #041624), GFAP (1:500, Dako, #Z0334), TUBA1A (1:200, Biorad, #MCAA776), microtubule-associated protein-2 (MAP2) (1:1,000 Merk-Millipore, #AB5622), and β-III tubulin (TUBB3) (1:1,000, Novusbio, #NB100-1612). The day after, cells were washed in PBS and then incubated in secondary antibodies combined as needed. Secondary antibodies Alexa Fluor 488 (1:500, Invitrogen, #A32723; Cell Signaling Technology, #4416), Alexa Fluor 568 (1:500, Invitrogen, #A11011 and #A11004), Alexa Fluor 647 (1:500, Invitrogen, #A31571), and 4′,6-diamidine-2′-phenylindole (DAPI) (1:500, Sigma, #32670) for nuclear staining were all diluted in blocking solution and were incubated onto fixed cells for 1 h at RT in the dark. The samples were finally mounted with a mounting medium (Sigma, #F6057).

### Animals

All experiments involving animals were carried out according to the directives of the Italian Ministry of Health regulating animal research (D.lgs 26/2014). CD1 mouse colonies were maintained at the Unit of Cell and Developmental Biology at the University of Pisa, in accordance with the requirements for animal experimentation (authorization #41/2019). C57BL/6J mice were utilized for glioma transplantation experiments. C57BL/6J mice were maintained in the animal facility of the National Council of Research (Pisa, Italy). All experimental procedures were conformed to the European Communities Council Directive #86/609/EEC and were approved by the Italian Ministry of Health (260/2016-PR, released on 3 November 2016).

### GL261 Cell Transplantation

A total of 3 C57BL/6J mice (22–27 g, age 8–10 weeks) were implanted with GL261 cells to induce the formation of glioma (Tantillo et al., 2020). Briefly, under a cocktail of ketamine/xylazine (100/10 mg/kg i.p.), mice received a stereotaxically guided injection of 40,000 GL261 cells (20,000 cells/μl in PBS solution) into the motor cortex (1.75 mm lateral to the midline, 0.5 anterior) with a Hamilton syringe guided by an automatized pump (Legato 130, Phymep) at a depth of 0.9 mm from the pial surface. Animals were kept for 23 days after cell implantation. For tissue collection, animals were deeply anesthetized with chloral hydrate and perfused transcardially with PBS followed by fixative (4% formaldehyde, 0.1 M sodium phosphate, pH 7.4). Brains were gently removed, post-fixed for 4 h in the same fixative at 4°C, cryoprotected by immersion in 30% sucrose, and cut using a sliding microtome (SM2010R, Leica) to obtain coronal sections of 50 μm thickness.

### Human Adult Brain Tissue Procurement

Human brain samples utilized in this study were supplied by Golgi-Cenci Foundation (Abbiategrasso, Italy) and were collected in line with its Human Research Ethics Committee. Specifically, the brain harvesting procedure was submitted and approved by the Ethics Committee of the University of Pavia on 6 October 2009 (Committee report 3/2009) and was conducted in accordance with the principles outlined in the Declaration of Helsinki of 1964 and its following amendments. All donors or next of kin or legal guardians received a consent form with the right to withdraw from the program at any time. Clinical information relative to each tissue was appropriately stored, and sample anonymity was warranted by a deidentifying numerical code.

### Human Brain Dissection

The preparation of human brain samples (4 from healthy subjects and 1 affected by GBM; age 79–80 years) was conducted, as previously described by Poloni et al. (2020). Briefly, autopsies were performed within 24 h after death, determined by an at least 20-min-long asystole. After the skull incision, cerebrum, cerebellum, brainstem, and rostral cervical spinal cord (first 3 metamers) were removed, separated, and then subjected to sections of about 1 cm. Tissue fixation was performed leaving slices in 10% phosphate-buffered formalin solution for 5 days at 4°C and washed in phosphate buffer for 2 days. Tissues were dehydrated in an automatic processor and embedded in paraffin using metal or plastic molds. Sections were finally cut using sledge and rotary microtomes (Leitz 1400, ARM3600, HistoLine Laboratories) into 8 μm thick sections.

### Glioblastoma Tissue Procurement

The GBM tissue collection was performed according to the Declaration of Helsinki with a protocol approved by the Ethics Committee of the University Hospital of Pisa (787/2015). Surgical resection was performed at the Neurosurgery Department of Livorno Civil Hospital (Livorno, Italy), and sample collection was conducted after informed consent. Patients’ anonymity was guaranteed by a de-identifying numerical code.

### Glioblastoma Multiforme Dissections

Additional GBM tissues were collected from patients undergoing tumor resection (*n* = 3) from the left temporoparietal lobe. Clinical diagnosis reported primary glioblastoma with no previous history of brain neoplasia. For histological analysis, brain tumor pieces were fixed in 10% formalin and embedded in paraffin. Sections were cut using a rotary microtome (Leica RM2245) into 4 μm-thick sections.

### Immunohistochemistry

Human brain sections were stewed at 58°C for 15 min and deparaffinized in xylene for 20 min. Rehydration was performed, incubating sections, for 10 min at a time, in ethanol solutions at decreasing percentages (100, 95, 80, and 70%). Samples were then washed in PBS and subjected to antigen retrieval at 121°C for 20 min in an automatic processor (2100 Retriever, BioVendor) using R-buffer A pH 6 (Electron Microscopy Science, #62706-10). After two additional washes in mQ water and three washes in PBS, sections were left in blocking solution (PBS supplied with 5% of horse serum and 0.3% of Triton X-100) for 1 h at RT in a humid chamber. Sections were then incubated with primary antibodies ON at 4°C. Primary antibodies were diluted in blocking solution as follows: CETN2 (1:500, Merck Millipore, #041624, clone 20H5, and 1:500, BioLegend, #W16110A), GFAP (1:500, Dako, #Z0334), ALDH1L1 (1:100, Abcam, #ab177463), S100β (1:100, GeneTex, #GTX129573), RNA-binding fox-1 homolog 3 (RBFOX3) (1:500, Merck-Millipore, #MAB377), ionized calcium-binding adaptor molecule 1 (IBA1) (1:250, Sigma, #SAB2702364), adenomatous polyposis coli (APC) (1:100, GeneTex, #GTX116009), platelet-derived growth factor receptor B (PDGFRB) (1:50, Abcam, #ab32570), CD34 (1:200, Abcam, #ab81289), and smooth muscle actin (SMA) (1:200, Abcam, #ab5694), aquaporin-4 (AQP4) (1:100, Abcam, #ab284135). Anti-CETN2 clone W16110A is used in Supplementary Figures 1A–C”, 2C–C”, 3E,E’. For all other sections, anti-CETN2 clone 20H5 was used. The day after, sections were washed three times in PBS and incubated in blocking solution supplemented with Alexa Fluor secondary antibodies (Invitrogen, #A11011, #A11004, and #A32723, Cell Signaling Technology, #4416, all diluted 1:500) and DAPI (1:500, Sigma, #32670). Sections were finally treated with an autofluorescence eliminator (Merck-Millipore, #2160) and washed with ethanol 70% for 3 min before being coverslipped with mounting medium (Sigma, #F6057). For Nissl staining, after deparaffinization with xylene and ethanol solutions in decreasing concentrations, sections were washed in deionized water and left in 0.1% cresyl violet solution for 5 min. Subsequently, sections were briefly washed with 0.1% acetic acid and finally with isopropanol. Sections were ultimately washed twice with xylene for 10 min at a time and coverslipped with mounting medium.

For the preparation of mouse samples, adult CD1 mice were euthanized, and brains and spinal cords were collected and fixed in 4% formaldehyde ON at 4°C. Brain samples were rinsed in PBS for 1 h and cryoprotected in 30% sucrose until they sank before being sectioned on a cryostat (CM1950, Leica) into 20 μm-thick sagittal sections. Spinal cords were cut in 20 μm-thick transverse sections. Sections were washed in PBS and left in blocking solution (PBS supplemented with 0.1% of Triton X-100 and 1% of horse serum) for 1 h at RT and then incubated with primary antibodies diluted in blocking solution, as reported earlier. The following day, primary antibodies were removed by a series of three washes in PBS followed by incubation with Alexa Fluor secondary antibodies and DAPI for nuclear staining, all diluted in blocking solution for 1 h at RT. Sections were coverslipped with mounting medium (Sigma, #F6057). Antibodies dilutions for human tissues were similarly applied onto murine sections.

Incubation with secondary antibodies alone was conducted as a control of staining quality.

### Imaging and Image Analysis

All images were acquired using an Olympus FluoView FV3000 confocal microscope. Image processing was performed using Olympus CellSens, Adobe Photoshop, and Adobe Illustrator. The co-localization study was performed with the co-localization threshold tool using the ImageJ software. The software allowed both the calculation of the Pearson’s correlation coefficient between two channels and their relative percentage of co-localization. Sections from 3 human brains were utilized for cell quantification.

## Results

### Centrin-2 Is a Centrosomal Protein in Human Neuroepithelial Stem Cells and Specifically Marks Differentiated Human Astrocytes

**FIGURE 1:**
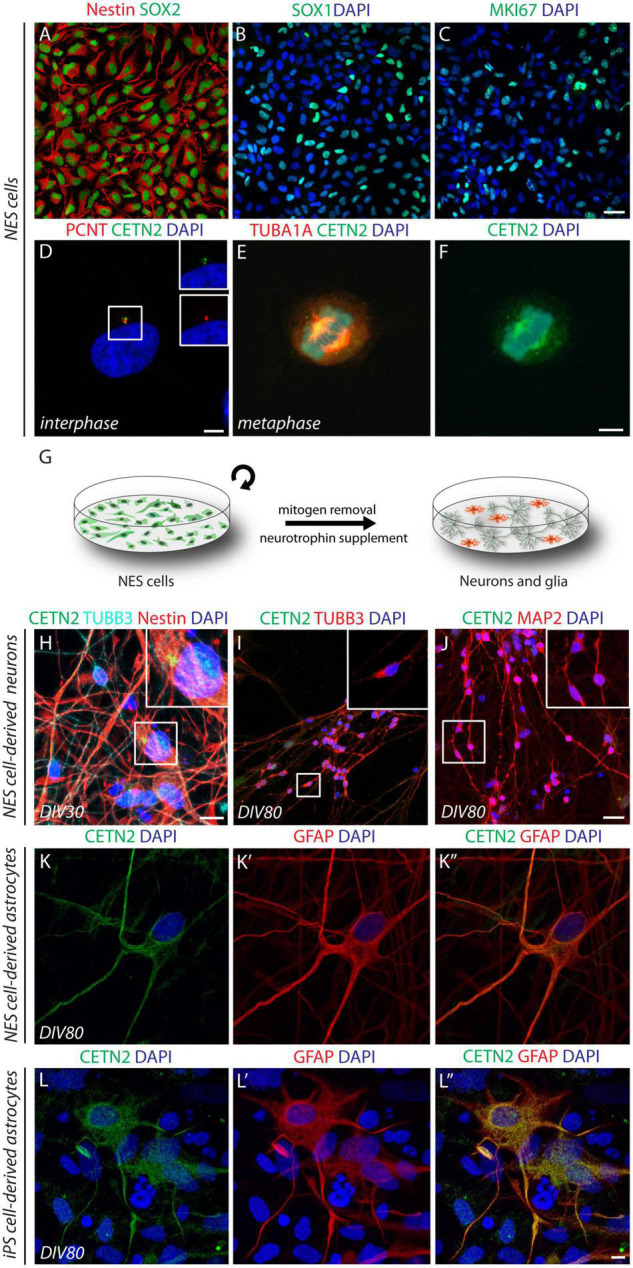
Cell-type specificity of centrin-2 (CETN2) expression in human neuroepithelial stem (NES) cells and induced pluripotent stem (iPS) cell progeny. **(A,B)** Human NES cells express canonical neural stem cell marker nestin and transcription factors SOX2 and SOX1. **(C)** Active cell proliferation is attested by MKI67 labeling. **(D–F)** CETN2 labels centrosomes and co-localizes with pericentrin (PCNT) during interphase, while it immunodecorates mitotic spindle poles during metaphase. **(G)** Graphical protocol for NES cell differentiation into mature neurons and glial cells. **(H)** Upon 30 days *in vitro* (DIV), NES-cell β-III tubulin (TUBB3)-labeled neuronal progeny is intermingled with nestin-positive NES cells, which retain centrosomal CETN2 expression (shown in inset). **(I,J)** NES cell-derived neurons at 80 DIV are immunopositive for TUBB3 and microtubule-associated protein-2 (MAP2). High magnification insets show no detectable centrosomal CETN2. **(K–K”)** NES cell-derived astrocytes at 80 DIV express cytoplasmic CETN2 co-localizing with the astrocytic marker glial fibrillary acidic protein (GFAP). **(L–L”)** Human iPS cell-derived astrocytes upon 80 DIV display similar cytoplasmic CETN2 localization and merge with GFAP staining. Scale bars: **(A–C)** 20 μm; **(D–F)** 5 μm; **(H)** 10 μm; **(I,J)** 20 μm; **(K,L”)** 5 μm.

To assess the CETN2 expression pattern in diverse cell types of the human brain, we took advantage of the NES cell system. Previously established neocortical NES cells (Onorati et al., 2016; Dell’Anno et al., 2018) were expanded in adhesion and propagated for up to 40 passages. NES cells expressed typical neural stem/progenitor markers as demonstrated through immunofluorescence analysis for the detection of intermediate filament protein nestin, present in the vast majority of cells (94.7 ± 3.4%, Figure 1A), as well as transcription factors SOX1 and SOX2 observed in 81.2 ± 6.1% and 98.8 ± 0.8% of cells, respectively (Figures 1A,B). Proliferation marker MKI67 immunopositivity was observed in 60.3 ± 2.5% of the cells, thus attesting active cell-cycle progression (Figure 1C). Immunofluorescence data revealed CETN2 centrosomal location during interphase, in co-localization with pericentrin (PCNT) (Figure 1D) and displacement at the poles of the mitotic spindle during metaphase, in all dividing NES cells (Figures 1E,F). These data support the self-renewing nature of NES cells and CETN2 dynamic profile coupled with cell-cycle progression.

We then investigated the expression profile of CETN2 during NES cell differentiation. Mitogens removal and exposure to neuronal differentiation medium supplemented with BDNF allow NES cell transition into mature neurons and glial cells, a procedure that lasts approximately 80 days *in vitro* (DIV) to achieve the expression of canonical neuronal and glial molecular markers (Figure 1G). Upon 30 DIV, TUBB3-positive newly born neurons were intermingled with undifferentiated nestin-positive NES cells where CETN2 expression in the centrosome was still appreciable (Figure 1H). After 80 DIV, 63.8 ± 6.6% of the cells were immunodecorated with mature neuronal marker MAP2 (Figure 1J). In NES cell-derived neurons, either at 30 DIV or 80 DIV, CETN2 was no longer detectable (Figures 1H–J), in agreement with centrosome loss of function as microtubule-organizing center (MTOC) in mature neuronal cells (Stiess et al., 2010; Kuijpers and Hoogenraad, 2011). Upon 80 DIV, NES cells also gave rise to GFAP-labeled astrocytes (2.7 ± 1% of total cells). Unexpectedly, about 1% of NES-cell derived astrocytes, identified by means of GFAP staining, exhibited CETN2 immunopositivity which appeared no longer condensed in the centrosome but was uniformly dispersed throughout the cytoplasm (Figures 1K–K”).

To assess whether similar observations could be collected in reprogrammed astrocytes, we exposed human iPS cells to a directed cerebro-cortical differentiation protocol (Chambers et al., 2009; Sousa et al., 2017). *In vitro* differentiation of iPS-derived progeny up to 80 days (DIV80) allowed the generation of GFAP-positive astrocytes and, similar to glial progeny obtained from NES cells, co-labeling with GFAP and CETN2 antibodies confirmed CETN2 expression in 20% of total astrocytes (Figures 1L–L”).

### Centrin-2 Is a Selective Astrocyte Molecular Marker

**FIGURE 2:**
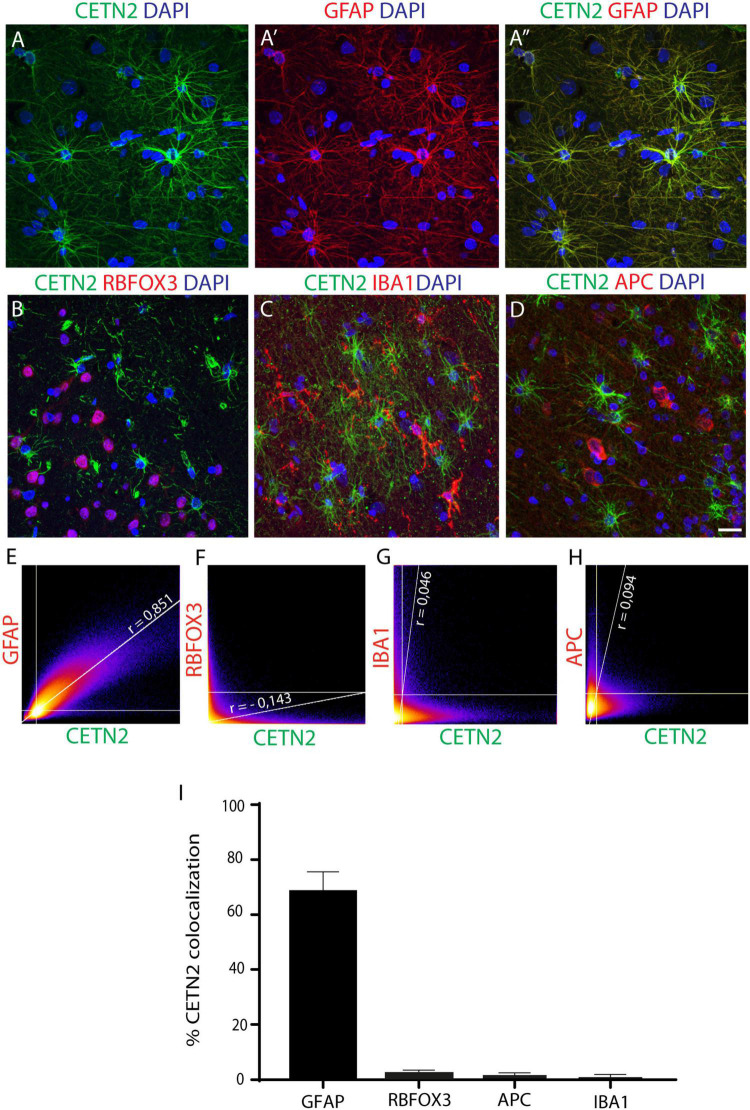
CETN2 specifically labels astrocytes. **(A–A”)** CETN2 labeling overlaps with canonical astrocytic marker GFAP in human prefrontal cortex sections. **(B–D)** CETN2 specificity is confirmed by a lack of co-localization with neuronal protein RNA-binding fox-1 homolog 3 (RBFOX3), microglial marker ionized calcium-binding adaptor molecule 1 (IBA1), and mature oligodendrocyte marker adenomatous polyposis coli (APC). **(E–H)** Scatterplot correlation graphs showing robust co-localization of CETN2 with GFAP and poor overlap with RBFOX3, as well as with IBA1 and APC. Pearson’s coefficients are indicated on each plot. **(I)** Histogram shows CETN2 co-localization percentages with other neuronal and non-neuronal cell types. Data represent mean ± SEM. Scale bar: **(A–D)** 20 μm.

Upon the initial findings in cultured astrocytes, we sought to validate CETN2 expression in human adult CNS. By incubating human cerebral cortex sections with the same antibody used on human NES cell progeny (clone 20H5), we observed that CETN2-labeling overlapped the GFAP signal (Figures 2A–A”). In contrast, we did not find CETN2 immunoreactivity in neurons, identified by means of RBFOX3 (also known as NeuN) labeling (Figure 2B) and, similarly, it did not label IBA1-positive microglia cells (Figure 2C) or APC-positive oligodendrocytes (Figure 2D). In agreement with this finding, we found that 80.7 ± 7.3% of GFAP-positive cells also expressed CETN2, with a Pearson’s correlation coefficient of 0.851 (Figures 2E,I). In contrast, no CETN2 signal was detected in neuronal (Figures 2F,I) or other non-neuronal cell types (Figures 2G–I).

CETN2 specificity in recognizing astrocytes was further validated by incubating human brain sections with another anti-CETN2 antibody (clone W16110A) which actually provided the same staining pattern (Supplementary Figures 1A–C”). The specificity of the signal was also corroborated by the overlap of CETN2 signal with other two known astrocytic markers, namely, ALDH1L1, and S100B (Supplementary Figures 1D–D”,E–E”). Nevertheless, compared with ALDH1L1 and S100B, whose signal appeared stronger in the perinuclear area, CETN2 exhibited a more uniform labeling of branches and branchlets, allowing the visualization of smaller astrocytic processes. The percentages of CETN2-labeled astrocytes also expressing ALDH1L1 and S100B immunoreactivity were quantified and were attested to be 30.4 ± 0.0% and 19.6 ± 0.0%, respectively (Supplementary Figure 1F). Overall, our results suggest that CETN2 labels uniquely astrocytic cells in the human brain.

### Centrin-2 Labels Protoplasmic and Fibrous Astrocytes in the Human Cerebral Cortex

**FIGURE 3:**
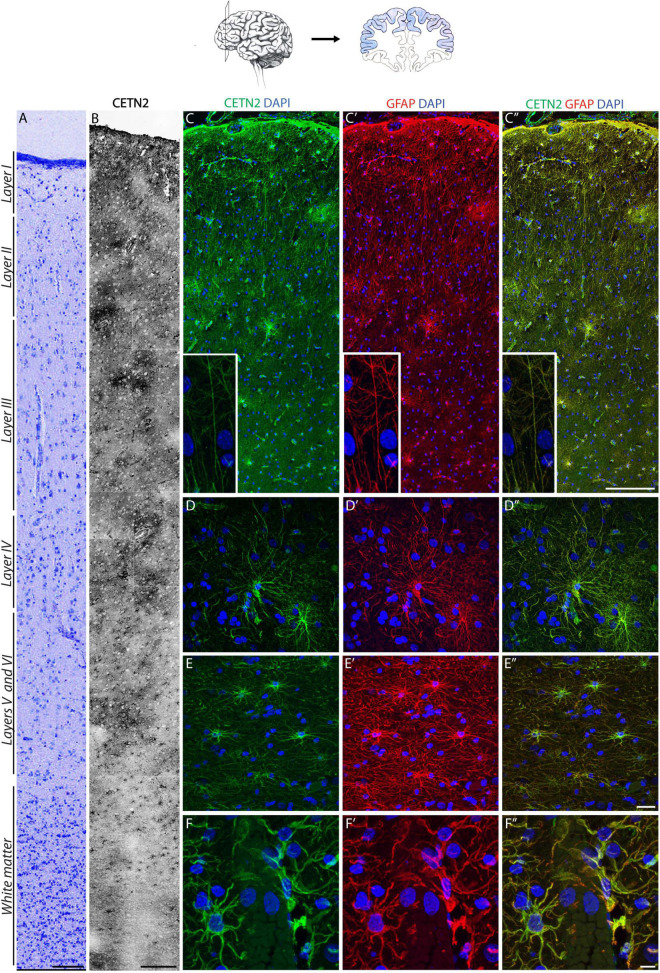
CETN2 labels protoplasmic and fibrous astrocytes in the human cerebral cortex. **(A)** Human prefrontal cortex colored with Nissl staining. **(B)** CETN2 labels astrocytes in human prefrontal cortex across all layers, in gray and white matter. **(C–C”)** Interlaminar astrocytes in layer I show CETN2 immunopositivity and co-labeling with GFAP. Cell bodies reside in layer I and extend numerous long processes with typical tortuous morphology (shown in inset). **(D–D”)** Protoplasmic astrocytes, largely abundant in cortical gray matter across layers II to VI, also exhibit CETN2 immunopositivity. **(E–E”)** Fibrous astrocytes in cortical white matter visualized by CETN2 and GFAP immunolabeling. **(F–F”)** CETN2 and GFAP immunolabeling of astrocytic end feet tightly surrounds cerebral blood vessel. Scale bars: **(A)** 100 μm; **(B)** 250 μm; **(C–C”)** 200 μm; **(D–D”),** and **(E–E”)** 20 μm; **(F–F”)** 10 μm.

Based on the above findings, we sought to perform a systematic analysis on the specificity of CETN2 expression in cortical layers. The cortical structure was first outlined by Nissl staining to label neurons across all layers (Figure 3A). Sections were then subjected to immunofluorescence analysis which revealed the presence of CETN2-positive astrocytes in the cerebral cortex, with a large abundance in upper layers (Figure 3B). In particular, CETN2 was clearly appreciable in layer 1 where interlaminar astrocytes reside. Labeling was detectable in processes adjacent to the pial surface as well as in the tortuous projections extending in a columnar manner through lower layers (Figures 3C–C”). Protoplasmic astrocytes detected in the gray matter of layers 2–6, with their typical dense arborization, also exhibited CETN2 immunopositivity in a pattern very similar to GFAP staining (Figures 3D–D”). In contrast, fibrous astrocytes in white matter displayed stronger CETN2 staining in somata and main branches and a progressively lighter signal in smaller processes (Figures 3E–E”).

Astrocytes are known components of the neurovascular unit and are part of the blood–brain barrier, mediating nutrient exchange from bloodstream to nerve cells (Simard and Nedergaard, 2004). In agreement with this function, CETN2-positive protoplasmic astrocytes were detected around vasculature with their end feet tightly juxtaposed onto the vessel surface (Figures 3F–F”). The polarized expression of AQP4 in astrocytes (Nagelhus et al., 2004) allowed the distinct visualization of end feet, which also appeared immunodecorated by CETN2 (Supplementary Figures 2A–A”). Observation of CETN2-staining pattern on vascular structures within the human brain obtained with two anti-CETN2 antibodies (clones 20H5 and W16110A) revealed that CETN2 was also identifying cellular components of the vascular wall that were not detectable by means of canonical GFAP staining (Supplementary Figures 2B–B”,C–C”). Counterstaining for CD34 revealed that CETN2 was labeling the innermost layer of the vessel, with CETN2 being mostly localized within endothelial cells cytoplasm and CD34 being expressed over their luminal membrane processes (Fina et al., 1990; Supplementary Figures 2D–D”). Smooth muscle cells, identified by means of SMA staining (Supplementary Figures 2E–E”), and pericytes recognized by the selective expression of PDGFRB (Supplementary Figures 2F–F”) did not exhibit CETN2 immunopositivity.

While the pattern of CETN2 labeling was very similar to GFAP in terms of diffuse intracellular localization, some differences have emerged in terms of areal distribution. In fact, unlike GFAP, CETN2 did not exhibit a uniform expression pattern across cerebro-cortical areas. For instance, CETN2 was detected in prefrontal (Figure 3) or parietal cortices (Supplementary Figures 3A,A’), whereas entorhinal cortex and hippocampal astrocytes were CETN2-negative (Supplementary Figures 3B,B’,C–C”).

### Centrin-2 Expression in Astrocytes of Other Anatomical Areas of Human Central Nervous System

Data collected from mouse CNS suggested the expression of CETN2 in ependymal cells lining the ventricles (Ying et al., 2014). We observed a similar pattern in human ependyma (Supplementary Figures 3D,D’), thus confirming the expression of CETN2 in CNS-ciliated cells. Then, we analyzed subcortical structures, including basal ganglia, before moving toward caudal segments.

**FIGURE 4:**
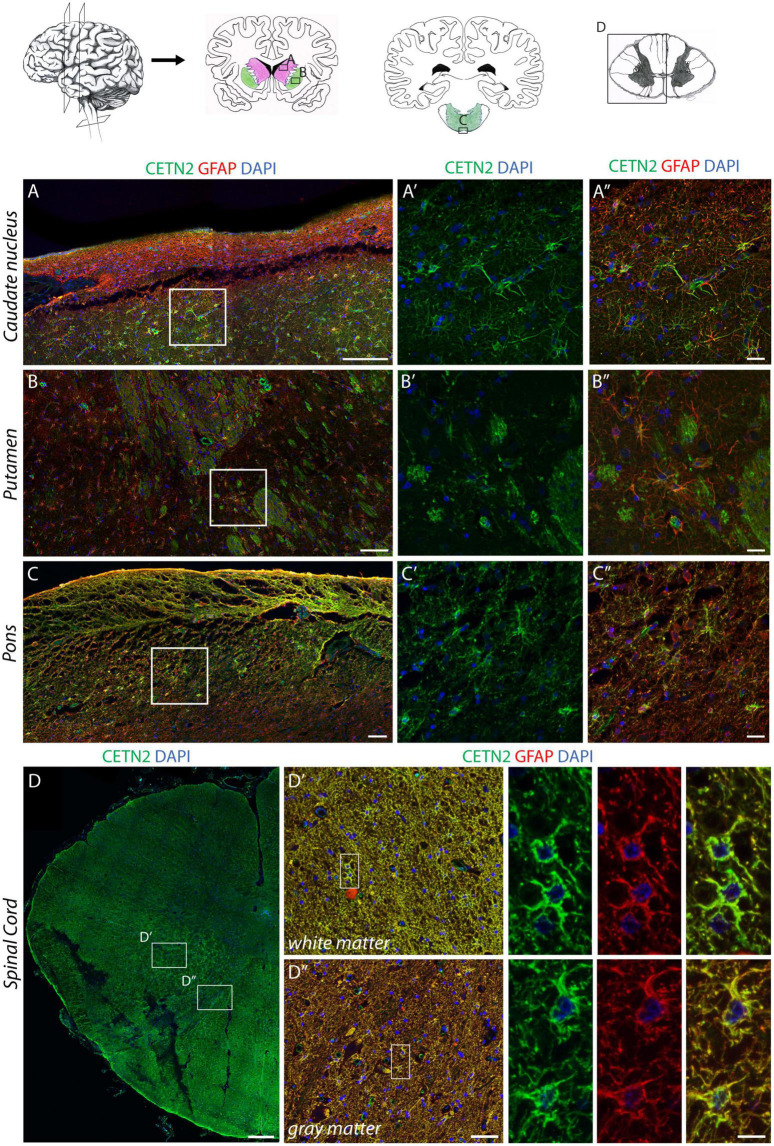
CETN2 expression in human central nervous system astrocytes. **(A–A”)** CETN2 staining in human subcortical structures. Astrocytes in caudate nucleus, identified by GFAP staining, also exhibit CETN2 immunopositivity. **(B–B”)** Astrocytes residing in the putamen show CETN2 expression as well as *capsula interna* fibers. **(C–C”)** GFAP-positive astrocytes in pons display CETN2 expression mostly detectable at the periphery of the tissue. **(D–D”)** Transverse section of the human cervical spinal cord with inserts shows high magnification details of gray and white matters, both exhibiting CETN2 expression in astrocytes. Scale bars: **(A–C)** 100 μm; **(A’,A”,B–B”,C’,C”)** 20 μm, **(D)** 500 μm; **(D’,D”)** 50 μm; inserts 10 μm.

Similar to the cerebral cortex, also the subcortical expression profile of CETN2 in diverse anatomical regions appeared uneven. For instance, astrocytes in the caudate nucleus exhibited CETN2 immunopositivity only in the medial portion close to the ventricle surface, whereas astrocytes in the lateral segment appeared CETN2-negative (Figures 4A–A”). In the putamen, CETN2 labeling provided a quite peculiar pattern highlighting resident astrocytes as well as the *capsula interna* (Figures 4B,B’). Since astrocyte projections are also known to envelope myelinated axonal fibers in proximity of Ranvier nodes (Black and Waxman, 1988), we performed a counterstaining with anti-neurofilament (NFL) antibody which allowed the visualization of axonal projections across putamen. Axonal segments appeared enveloped by CETN2-positive structures arguably referred to as perinodal astrocytes’ extroflexions (Supplementary Figures 3E,E’).

In the cerebellar cortex, astrocytes were only identified by means of canonical GFAP staining (Supplementary Figures 3F,F’). In contrast, fibrous astrocytes in the *arbor vitae* provided a clear immunopositivity for CETN2 (Supplementary Figures 3G–G”). The dorsal portion of pons also exhibited CETN2 immunopositivity (Figures 4C’,C”) as well as the spinal cord where CETN2 signal was detected not only in central canal-ciliated cells (Supplementary Figures 3H–H”), but also uniformly in white and gray matter (Figures 4D–D”).

Overall, data collected from a systematic investigation of CETN2 expression along the rostro-caudal axis demonstrated that CETN2-positive astrocytes are distributed in diverse anatomical areas of the human CNS with a patched distribution and larger abundance in the cerebral cortex and spinal cord.

### Centrin-2 Is Poorly Expressed in Mouse Astrocytes

Previous reports aimed at investigating primary cilia in neurons and astrocytes during neurodevelopment or in response to insults have utilized CETN2-GFP transgenic mouse lines (Bangs et al., 2015; Sterpka et al., 2020). In these studies, authors focused on rostral CNS structures and on early embryonic neurodevelopmental stages, without describing CETN2 immunopositivity in astrocytes. This observation raised the question of whether CETN2 had a unique expression in human nervous tissue. Therefore, we conducted a systematic analysis to assess CETN2 expression along the anteroposterior axis of murine CNS from the forebrain to the most caudal portion of the spinal cord. Transcriptomics analysis conducted on the human and mouse brains suggested an upregulation of *CETN2* in human astrocytes compared to their murine counterparts (Zhang et al., 2016), and in agreement with this report, CETN2 was scarcely observed in mouse CNS. With the only exception of ciliated cells of the ependyma (Supplementary Figures 4A,B,B’) and of spinal cord central canal (Supplementary Figures 4C,D,D’), scarce CETN2-positive astrocytes were observed in spinal cord white matter (Supplementary Figures 4C,E,E’) and in the pons (Supplementary Figures 4F,G,G’,H,H’), whereas all other investigated areas, including cortex, corpus callosum, hippocampus, striatum, and cerebellum astrocytes, were exclusively identified by means of GFAP staining (Supplementary Figure 5).

The complete absence of CETN2 in mouse rostral CNS structures, compared to results collected from human specimens, suggests that CETN2 expression in astrocytes is a peculiar trait of the human brain.

### Centrin-2 Labels Neoplastic Astrocytes in Glioblastoma Multiforme

Astrocytes play a major role in the evolution of many common primary brain tumors, gliomas, and a less prominent, though significant, role in the progression of metastases. From a practical diagnostic viewpoint, GFAP is a reliable immunohistochemical marker for staining surgically resected brain neoplasms to ascertain whether they have a significant component of cells differentiating along the astrocytic pathway (Sofroniew and Vinters, 2010). Several centrosomal genes have also been reported to have an abnormal expression in brain tumors. Among these, *CETN2* mRNA has been described to be overexpressed in astrocytomas and in its most aggressive form, GBM (Loh et al., 2010, 2012; Lu et al., 2020). However, at present, no indications have been provided to describe CETN2 cellular localization in neoplastic tissue.

**FIGURE 5:**
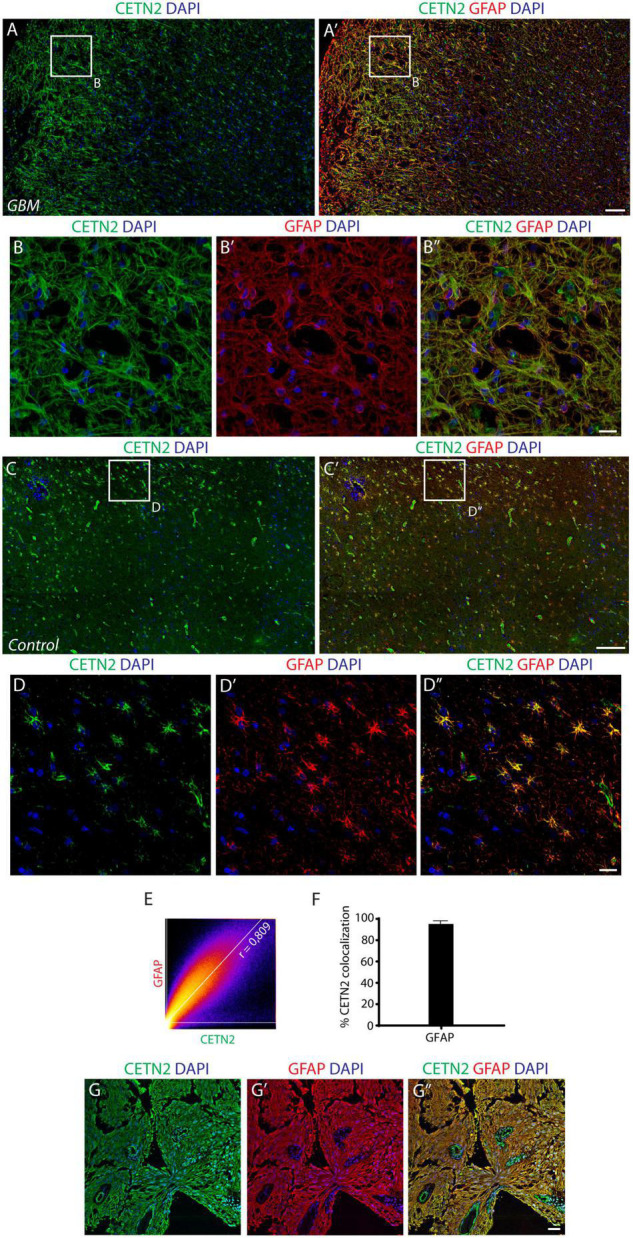
CETN2 expression in glioblastoma multiforme (GBM). **(A)** Human cerebral cortex affected by GBM. Neoplastic astrocytes, magnified in **(B–B”)**, are immunolabeled by CETN2 and GFAP and appear densely clustered in the lesion core. **(C–C’)** In healthy human cerebral cortex, conversely, CETN2-positive astrocytes are uniformly dispersed in the parenchyma. **(D–D”)** High magnification images of healthy CETN2-positive astrocytes also display GFAP immunoreactivity. **(E)** Scatterplot correlation graph shows robust co-localization of CETN2 and GFAP in human GBM. **(F)** Histogram representing the percentage of GFAP-positive astrocytes displays CETN2 immunopositivity in GBM. Data show mean ± SEM. **(G–G”)** Surgically resected GBM stained for CETN2 and GFAP. Astrocytes are tightly intermingled in the tumor and display immunopositivity for both CETN2 and GFAP. Unlike GFAP, CETN2 staining also allows vessel visualization. Scale bars: **(A,A’, C,C’)** 100 μm; **(B–B”, D–D”, G–G”)** 20 μm.

On the basis of our observations, we sought to investigate the expression of CETN2 in a human brain containing GBM and in primary GBM tissues collected after surgical resection. Astrocytes within GBM exhibited a strong immunopositivity for CETN2 (Figures 5A–B”) and appeared tightly clustered in the lesion core compared to the dispersed distribution they present in healthy cerebral parenchyma (Figures 5C–D”). In all our samples collected after surgical GBM resection, CETN2 was found to be expressed in astrocytes with a percentage of co-localization of CETN2 and GFAP was estimated at 95.0 ± 2.2% (Figures 5E,F). Similar to healthy tissues, vascular endothelium also appeared CETN2 positive (Figures 5G–G”).

To confirm CETN2 expression in GBM, we also examined murine GBM cells (GL261) both *in vitro* and *in vivo*. Immunofluorescence assay confirmed the expression of CETN2 in cultured GL261 cells as well as upon transplantation in the mouse brain, although, in the latter case, the expression appeared sparser than human GBM (Supplementary Figure 6). Collectively, these findings support the validity of CETN2 as a novel marker of brain tumors.

## Discussion

Centrosomal protein CETN2 has been described to absolve relevant functions in eukaryotic cells: it assists centriole duplication (Salisbury et al., 2002), and it guarantees the functioning of ciliary basal bodies (Dantas et al., 2012). In addition, it participates in non-centrosomal functions, such as NER, a process that is fundamental to ensure genome stability (Dantas et al., 2012). CETN2 is known for being expressed in mature cells of the CNS, specifically photoreceptors and ependymal cells (Ying et al., 2014). Nevertheless, no information is currently available about its expression profile in human neural stem cells and their progeny. NES cells possess a great self-renewing potential and a wide multilineage differentiation ability and represent an ideal platform for *in vitro* studies related to neural stem cell biology, neuronal, and glial differentiation. Taking advantage of this cellular system, we explored CETN2 expression pattern in NES cells and their differentiation product, demonstrating, for the first time, the selective presence of CETN2 in human astrocytes.

Glia represents about 50% of cell types in the CNS (Barres, 2008), and astrocytes, in particular, are known to absolve multiple functions, including contributions to the blood–brain barrier, synaptogenesis, neuronal circuitry, ion homeostasis, neurotransmitter buffering, secretion of neuroactive agents, and response to harmful events (Khakh and Deneen, 2019). GFAP is the most well-known astrocyte molecular marker (Eng et al., 2000), but accumulating evidence has shown that GFAP alone poorly represents the entire astrocyte population in CNS, whose molecular diversity across anatomical areas, or in response to insults, requires a combination of different molecular markers (Khakh and Deneen, 2019). Indeed, GFAP labeling is often complemented by an array of supplemental proteins ranging from gap junction molecules (i.e., connexin 30), adhesion molecules (i.e., CD44), enzymes (i.e., ALDH1L1 and glutamine synthetase), transporters (i.e., GLAST), or ion channels (KCNJ10, also known as Kir4.1), mostly depending on the anatomical area under investigation (Lehre et al., 1995; Nagy et al., 1999; Poopalasundaram et al., 2000; Sosunov et al., 2014; Verkhratsky and Nedergaard, 2018; Khakh and Deneen, 2019).

The extreme diversity of astrocytes in morphological and molecular terms across the CNS rostro-caudal axis also implies functional heterogeneity (Khakh and Sofroniew, 2015). A key element to diversity is associated with calcium signals that play a critical role in a wide number of physiological and pathological astrocyte functions (Khakh and Sofroniew, 2015). Although investigations conducted on murine tissues suggest the presence of several calcium-binding proteins in astrocytes (Chai et al., 2017), S100B is currently the only calcium-chelating protein conventionally used as a specific astrocyte marker. Both S100B and CETN2 belong to the large group of EF-hand calcium-binding proteins, but unlike S100B, which is also expressed in oligodendrocytes (Heizmann et al., 2002), we report CETN2 as selectively expressed in astrocytic cells.

We observed CETN2 expression in diverse anatomical areas and, with the exception of the spinal cord, where the distribution appeared uniform in gray and white matter, CETN2-positive astrocytes exhibited a rather clustered arrangement. In these areas, CETN2-positive astrocytic end feet enveloped vessels, which also expressed CETN2 in correspondence of endothelial cells. Calcium mobilization is a key element in vasoregulation, not only in astrocytes where its intracellular increase has been linked to functional hyperemia (Takano et al., 2006), but also in endothelial and smooth muscle cells, where it plays a role in both contraction and relaxation (Brenner et al., 2000). Since cerebral blood flow is increased within localized regions of activity following neuronal stimulation (Watts et al., 2018), the expression of CETN2 in discrete areas may suggest a possible role for this protein in modulating blood flow at the gliovascular interface. Our data also show that the distribution of CETN2-positive astrocytes appeared uneven across diverse cortical areas. In fact, CETN2-positive astrocytes were more represented in the isocortex (prefrontal and parietal neocortex), rather than in meso- and allocortex (entorhinal and hippocampal areas) (Puelles et al., 2019), thus suggesting a larger abundance of this marker in the newest and more evolved segments of the human cerebral cortex. Our investigation on cell-type-specific expression of CETN2 is also corroborated by transcriptomic studies on the human brain. In particular, Li et al. (2018) analyzed at single-cell resolution the transcriptional traits across multiple human brain regions ranging in age from embryonic development through adulthood. We interrogated the dataset for *CETN2*, and during development, it appears in neural stem/progenitor cells and in nascent neurons and astrocytes (Li et al., 2018). By single-nucleus RNA sequencing, *CETN2* is also reported in the adult brain, in particular in astrocytes, even if CETN2 does not appear to be a selective marker, since other cell types may express the gene (e.g., proliferating cells). Our study demonstrates the importance of protein expression validation into multiple cell/tissue sources, from *in vitro* systems to brain specimens, to correctly profile the expression and localization of a protein marker.

Human astrocytes have been reported to present several differences compared with their murine counterparts encompassing morphological features, size, and transcriptional profile (Oberheim et al., 2009; Zhang et al., 2016). In our work, we pointed out that CETN2-positive astrocytes are drastically more prevalent in humans, rather than in mouse CNS. In particular, this appears to be most evident in the mouse cerebral cortex, where no CETN2 expressing astrocytes were detected. This finding indicates that the evolution of the human cortex with its increase in size and complexity, compared with the lissencephalic mouse brain, may have positively selected CETN2 astrocytic expression. Whether this may be due to increased energy consumption, and consequently to a finer modulation of cerebral blood flow in the human brain compared with the mouse brain, still needs to be determined.

Astrocytes are also known to be highly interconnected and endowed with a surprising ability to move and modify their functional state in specific physiological or pathological conditions (Lagos-Cabre et al., 2019). Indeed, astrocyte migration plays a major role in glioma spreading, leading to infiltration of normal tissue (Qi et al., 2017). Based on these considerations, we also explored CETN2 expression in GBM as a prototypical tumor with glial overgrowth. In GBM, channels involved in ionic transport have their expression frequently affected (Polisetty et al., 2012) and calcium concentration, in particular, has been demonstrated to be pivotal for tumor initiation, maintenance, and infiltration (Leclerc et al., 2016; Guan et al., 2018). Accordingly, the transcriptional profile of several genes coding for calcium-binding proteins, including *CETN2*, have their expression altered in GBM (Loh et al., 2010, 2012; Robil et al., 2015; Lu et al., 2020). Our results demonstrate that CETN2 is expressed in astrocytes of primary glioblastomas, in a stage of tumor progression which is, by definition, the most severe. Lu et al. (2020) also reported that low *CETN2* expression could be correlated to better survival of patients with GBM, thus suggesting that *CETN2* expression increases with disease evolution. However, a more systematic investigation on lower grade astrocytomas would be needed to validate this hypothesis.

GBM spreading has been shown to follow peculiar pathways, leaving substantially some unaffected anatomical areas (i.e., hippocampus) (Mughal et al., 2018). Given the role of calcium signaling in GBM infiltration, a role of CETN2 in determining the susceptibility of specific regions to cancer growth cannot be excluded. In addition, the presence of CETN2-positive cells in vessels within cancer may reinforce our speculation over CETN2 in affecting blood flow, a parameter that may be relevant to tumor growth. However, the question of whether in neoplastic astrocytes CETN2 triggers tumor initiation, a function that is mostly related to its involvement in DNA repair, or tumor growth, by affecting intracellular calcium signaling or angiogenesis will be subject of future investigations.

## Conclusion

In conclusion, the identification of CETN2 as a novel astrocyte molecular marker adds a new title for a comprehensive understanding of astrocyte heterogeneity and function in healthy CNS and disease. Moreover, it contributes to future investigations on specific astrocyte biology with an impact on human brain function and disease.

## Data Availability Statement

The raw data supporting the conclusions of this article will be made available by the authors, without undue reservation.

## Ethics Statement

The animal study using CD1 mice was reviewed and approved by the OPBA of the University of Pisa (authorization #41/2019). C57BL/6J mice, used for transplantation experiments, were maintained in the animal facility of the National Council of Research (Pisa, Italy). All experimental procedures were conformed to the European Communities Council Directive #86/609/EEC and were approved by the Italian Ministry of Health (260/2016-PR, released on November 2016).

## Author Contributions

ED’I performed immunohistochemistry on mouse and human sections and completed scatterplot analysis and relative quantifications. TP and VM dissected human brains, prepared paraffin-embedded sections, contributed to data collection and analysis, and assisted in the writing of the text. MO provided human NES cells. UB provided murine tissues. LR performed immunofluorescent assays *in vitro* and relative quantifications. EV cultured GL261 cells and performed transplantation experiments. MO and CD’A supplied iPS cell-derived astrocytes and relative analysis. MD’A conceived the experiments, expanded and differentiated NES cells, performed immunohistochemistry and immunocytochemistry procedures, acquired all confocal images, and drew anatomical brain sections. ED’I and MD’A prepared figures and wrote the manuscript. All authors provided intellectual content and critical review of the manuscript.

## Conflict of Interest

The authors declare that the research was conducted in the absence of any commercial or financial relationships that could be construed as a potential conflict of interest.

## Publisher’s Note

All claims expressed in this article are solely those of the authors and do not necessarily represent those of their affiliated organizations, or those of the publisher, the editors and the reviewers. Any product that may be evaluated in this article, or claim that may be made by its manufacturer, is not guaranteed or endorsed by the publisher.
