# Supplementary material for: Centrin 2: A Novel Marker of Mature and Neoplastic Human Astrocytes
Source: Front Cell Neurosci. 2022 Apr 29;16:858347. doi: 10.3389/fncel.2022.858347 (PMC9100563; doi:10.3389/fncel.2022.858347)
Supplement: Supplementary file 1 [file Data_Sheet_1.docx]

Supplementary Material


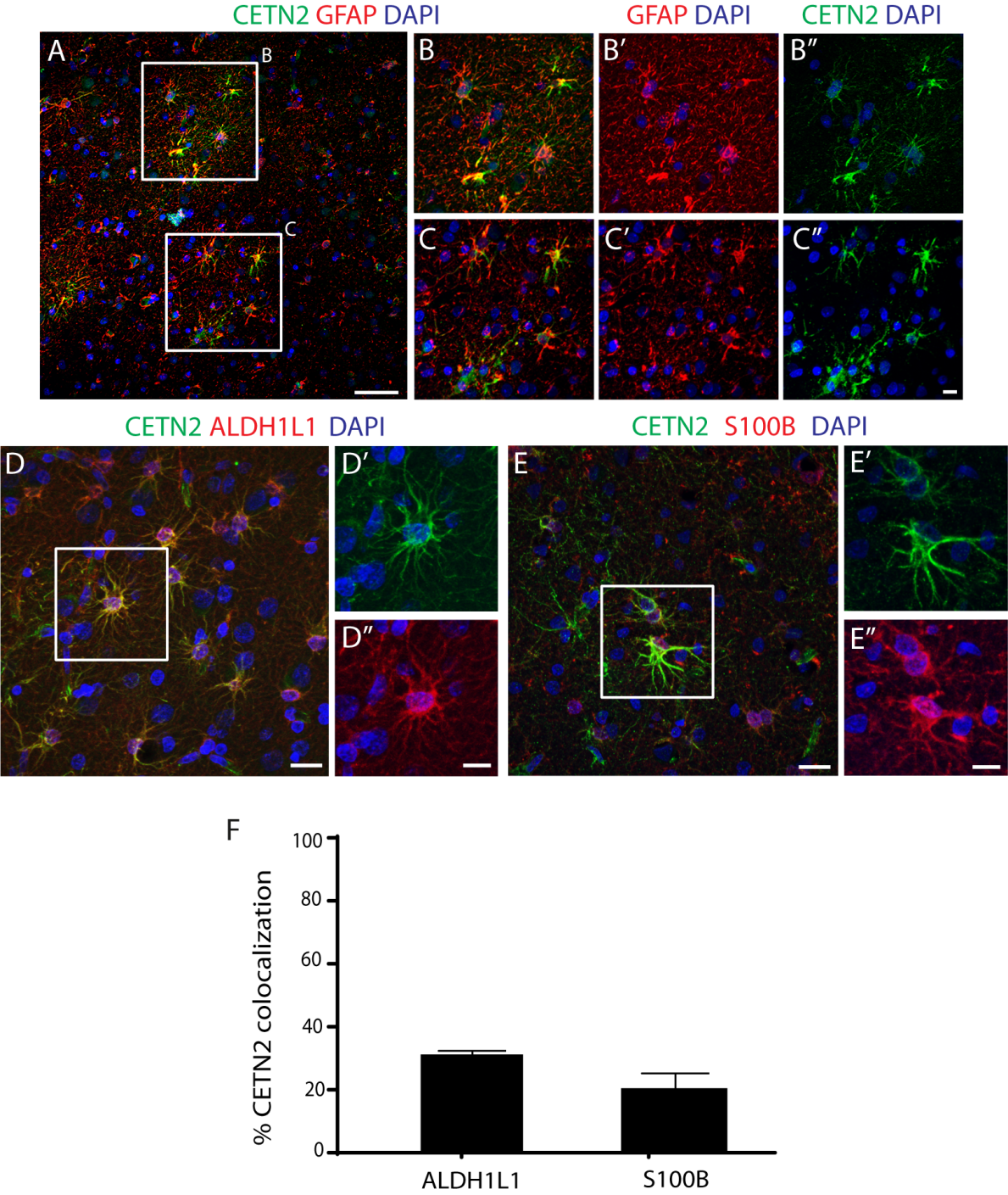


**Supplementary Figure 1. Validation of CETN2 immunostaining pattern.**

**(A-C’)** Astrocytes in human cortex can also be efficiently labelled by using the anti-CETN2 antibody (clone W16110A), whose pattern overlaps GFAP staining. (**D-D’’**) Similarly, CETN2 signal merges with ALDH1L1 staining. (**E-E’’**) CETN2 signal on human adult astrocytes shown in double-staining with S100B. **(F)** Histogram showing the percentage of CETN2-labelled astrocytes also expressing known astrocytic markers ALDH1L1 and S100B. Data represent mean ± SEM. Scale bars: A, D and E 20 µm, B-B’’, C-C’’, D-D’ and E-E’10 µm.


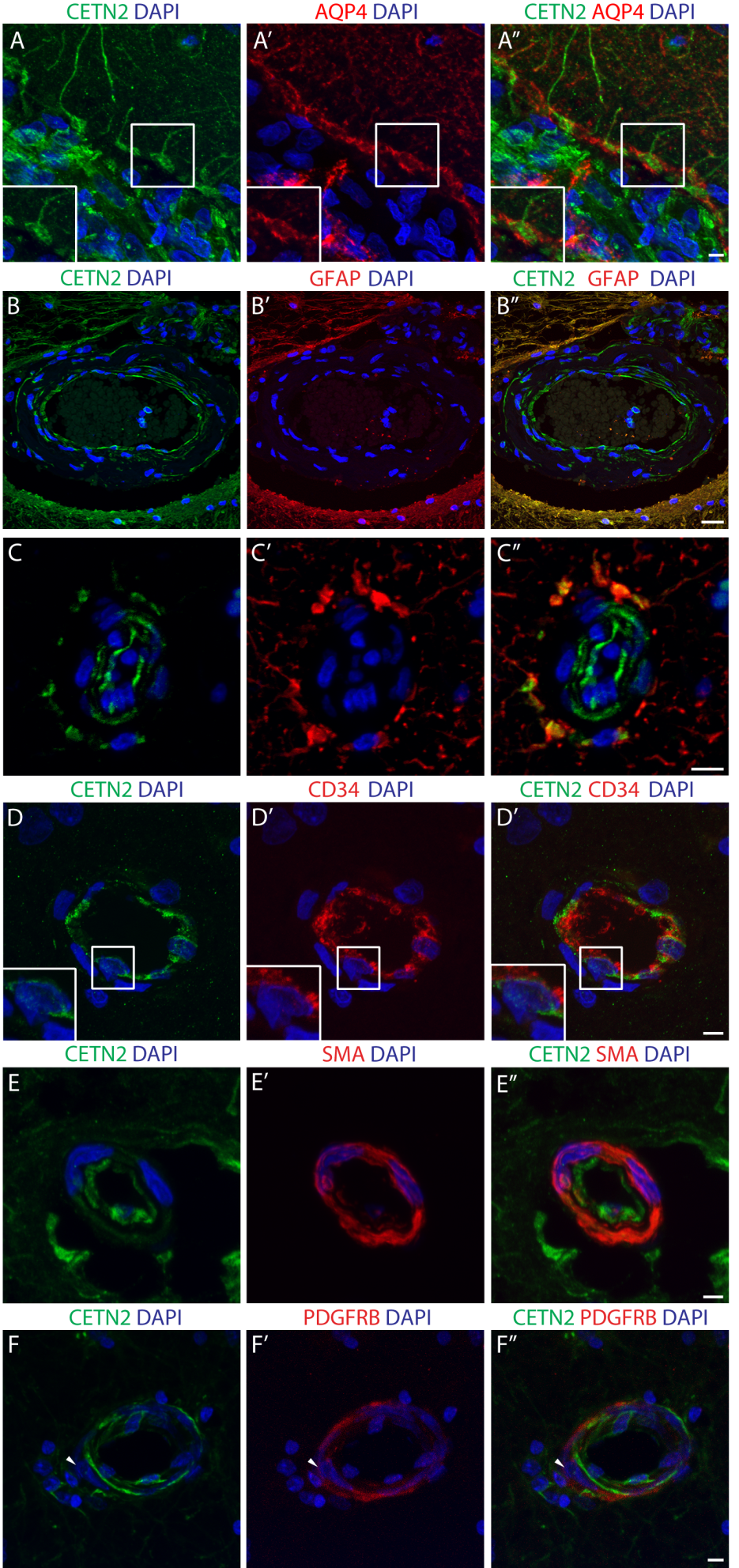


**Supplementary Figure 2. CETN2 expression in the gliovascular unit. (A-A’’)** CETN2-positive astrocytic end feet surrounding the coronal section of a vessel are immunodecorated by AQP4. **(B-B’’)** Unlike GFAP, anti-CETN2 antibody (clone 20H5) also labels cells within the vascular wall. **(C-C’’)** Similarly, the anti-CETN2 antibody (clone W16110A) exhibits a comparable pattern. **(D-D’’)** Counterstaining for CD34 allows the identification of endothelial cells. CD34 labels the luminal membrane of endothelial cells, whose cytoplasm displays CETN2 immunoreactivity. **(E-E’’)** Larger caliber vessel stained for SMA and CETN2. Unlike endothelium, which retains a clearly detectable CETN2 signal, smooth muscle cells do not appear CETN2-immunoreactive. **(F-F’’**) Pericytes envelope the abluminal surface of endothelium and are PDGFRB immunoreactive but do not express CETN2. White arrow indicates pericyte nucleus. Scale bars: A-A’’5 µm, B-B’’ 20 µm, C-C’’ 10 µm, D-F’’5 µm.


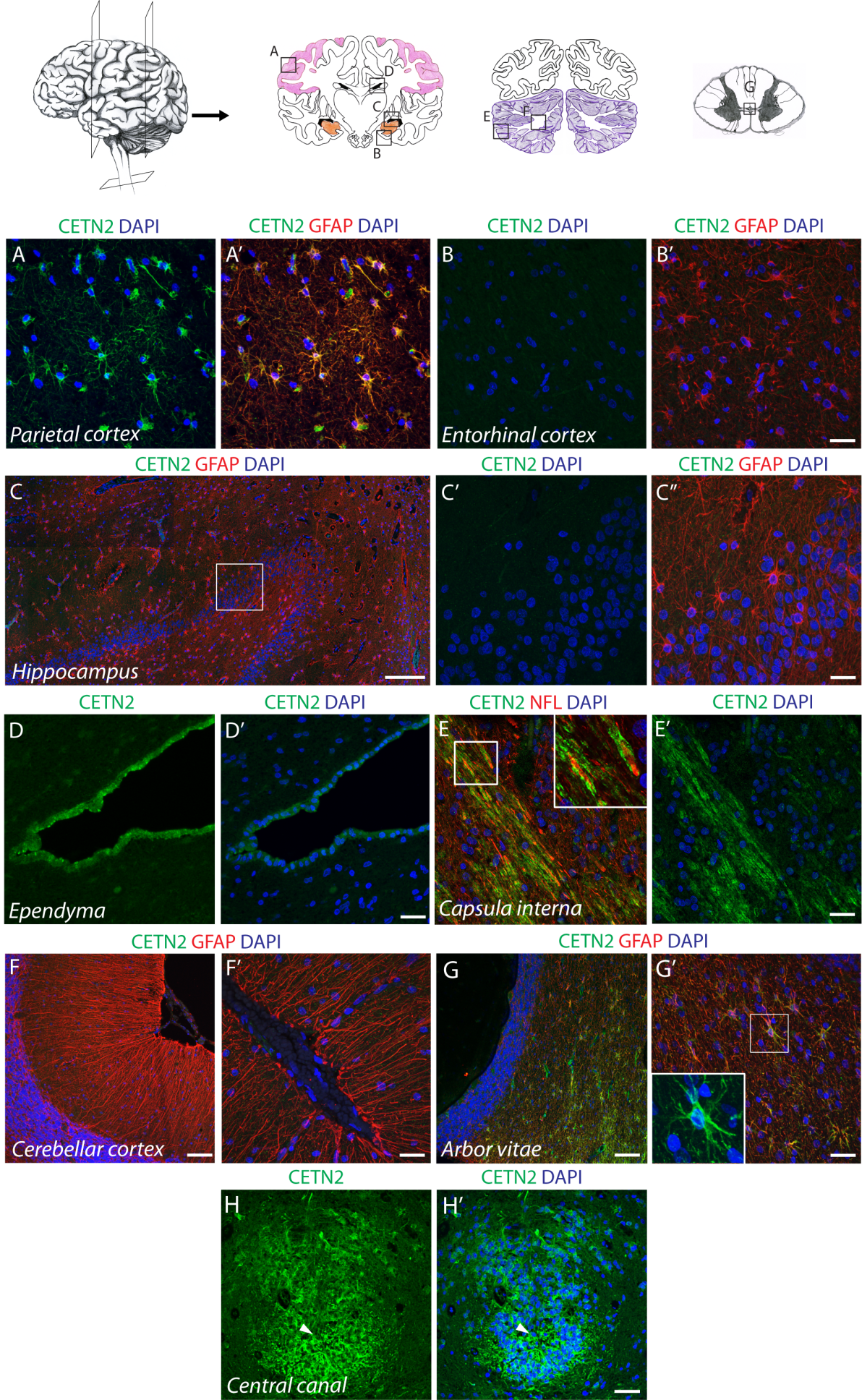


**Supplementary Figure 3. Selective expression of CETN2 in astrocytes of the CNS.**

**(A-A’)** Detail of human parietal cortex showing the presence of CETN2-positive astrocytes. **(B-B’)** Detail of human entorhinal cortex showing no expression of CETN2-positive astrocytes. **(C-C’’)** Hippocampal astrocytes are CETN2 negative. **(D-D’)** Ependymal cells of the lateral ventricle exhibit CETN2 expression. **(E-E’)** *Capsula interna* detail counterstained for NFL showing axonal projections surrounded by CETN2 immunoreactive astrocyte projections. **(F-F’)** Unlike cerebral cortex, cerebellar cortex shows no presence of CETN2 immunodecorated astrocytes, which, instead, are present in the cerebellum white matter **(G-G’)**. **(H-H’)** Spinal cord central canal, indicated by the white arrow, exhibiting CETN2-positive cells lining the lumen. Scale bars: A-B’ 20 µm; C 200 µm; C’-C’’ 20 µm; D-D’ 20 µm; E 10 µm; E’ 20 µm; F 10 µm; F’ 20 µm; G-G’ 20 µm.


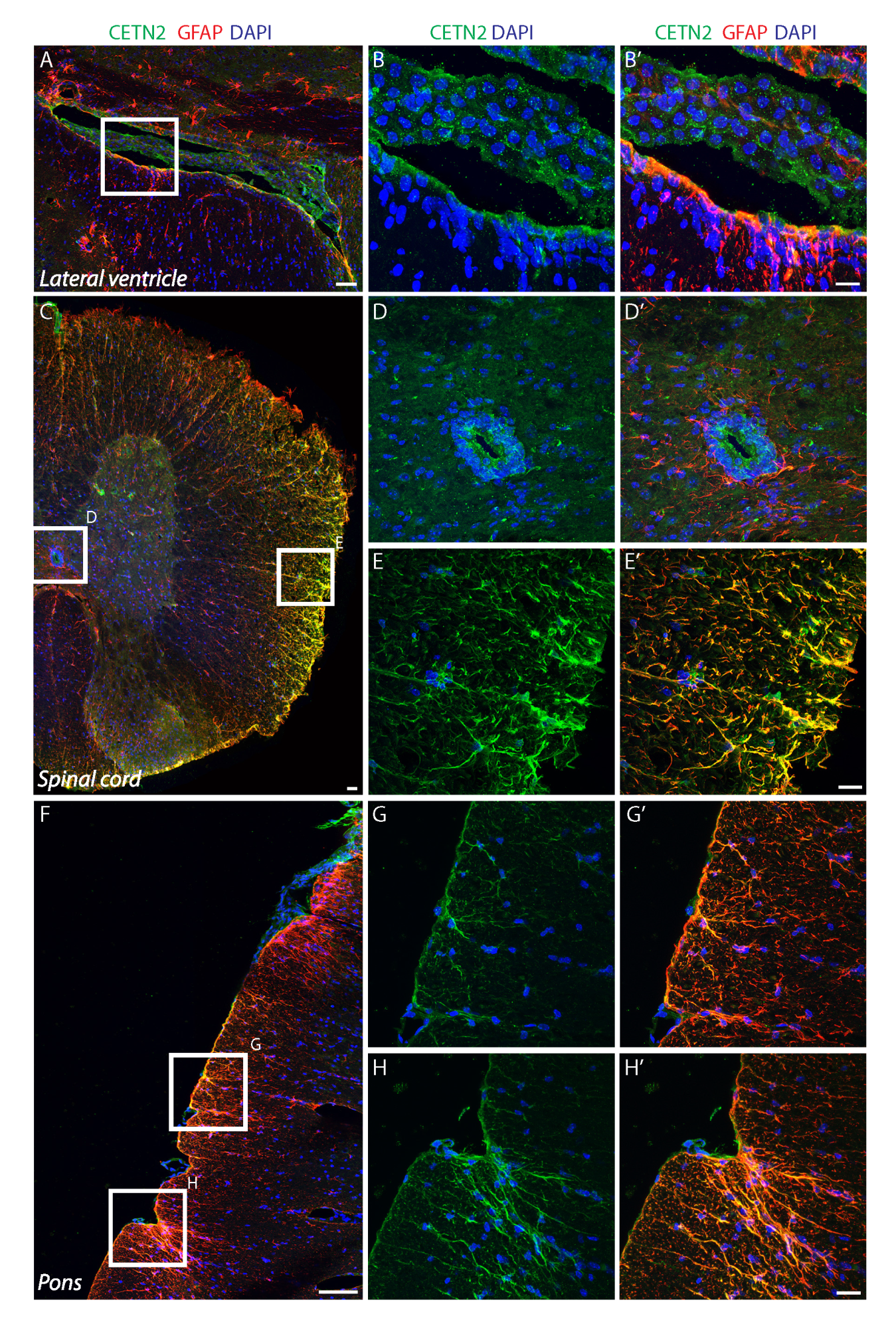


**Supplementary Figure 4. CETN2 expression pattern in mouse CNS.**

**(A)** CETN2-positive ependymal cells lining adult lateral ventricle in mouse. **(B-B’)** High magnification of CETN2 labeling and counterstaining with GFAP. **(C)** Transverse section of mouse spinal cord showing CETN2 expression in white matter astrocytes and in central canal. **(D-D’)** High magnification of spinal cord central canal displaying CETN2-positive cells. **(E-E’)** Spinal cord white matter CETN2-positive astrocytes and merge with GFAP signal. **(F)** Low magnification picture of mouse pons showing the presence of few CETN2-positive cells in the parenchyma. **(G-G’;** **H-H’**) High magnification pictures of insets in (F) showing the astrocytic nature of CETN2-positive cells. Scale bars: A, C and F 100 µm; B-B’, D-D’, E’E’, G-G’ and H-H’ 20 µm.


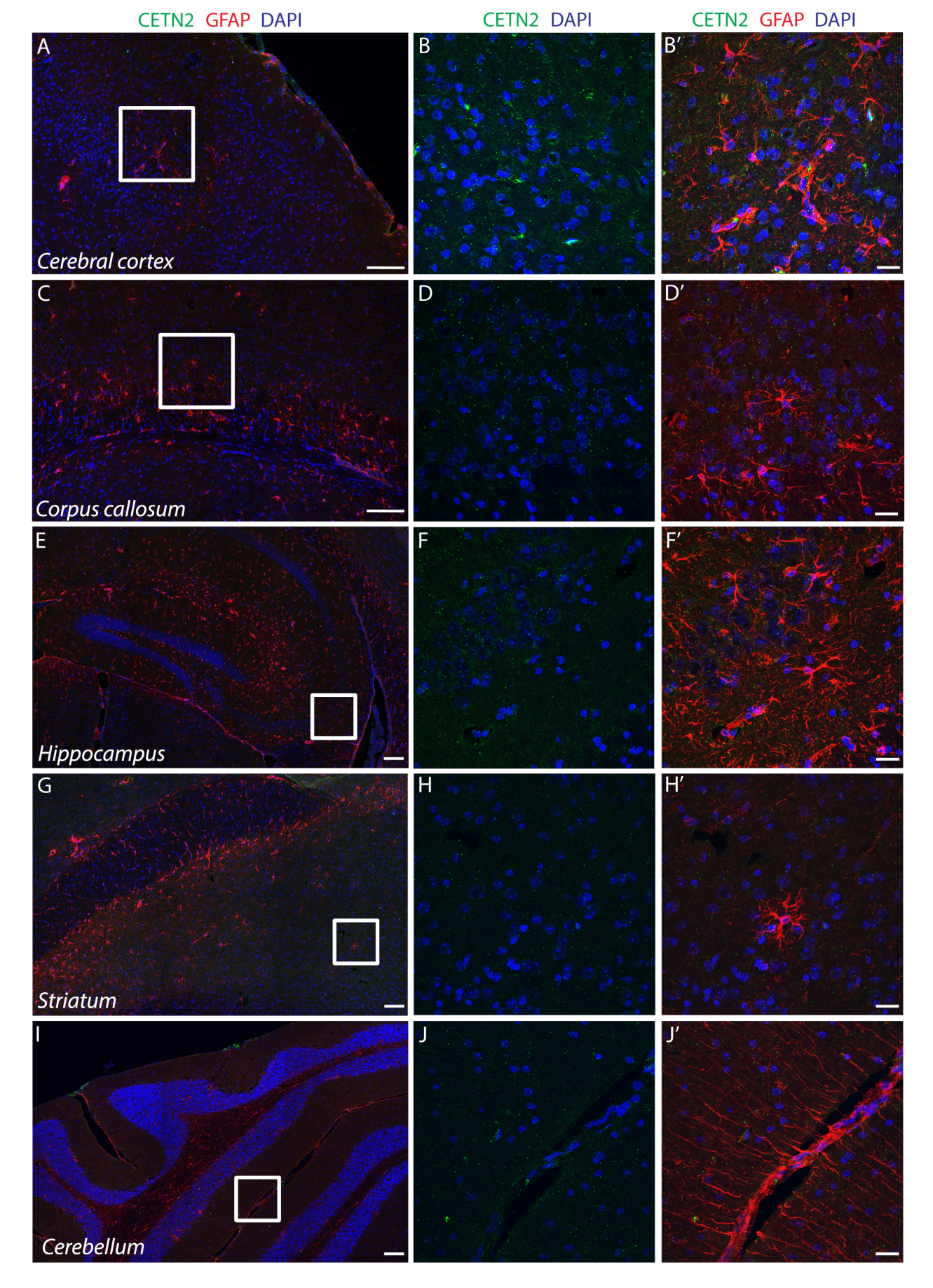


**Supplementary Figure 5. CETN2-labelled astrocytes are scarcely represented in mouse CNS.**

**(A)** Unlike human cerebral cortex, murine cortical tissue shows no presence of CETN2-labelled astrocytes. **(B-B’)** High magnification images of cortical astrocytes exclusively displaying GFAP expression. **(C)** Mouse corpus callosum astrocytes do not express CETN2. **(D-D’)** High magnification images of corpus callosum double-stained for CETN2 and GFAP. **(E)** Similarly to human, murine hippocampus shows no CETN2-positive astrocytes. **(F-F’)** High magnification of hippocampal astrocytes stained for CETN2 and GFAP. **(G, H-H’)** Few astrocytes were found in the corpus striatum, exclusively displaying GFAP-immunopositivity. **(I-J’)** Cerebellar cortex astrocytes are CETN2-negative. Scale bars: A, C, E, G and I 100 µm; B-B’, D-D’, F-F’, H-H’ and J-J’ 20 µm.


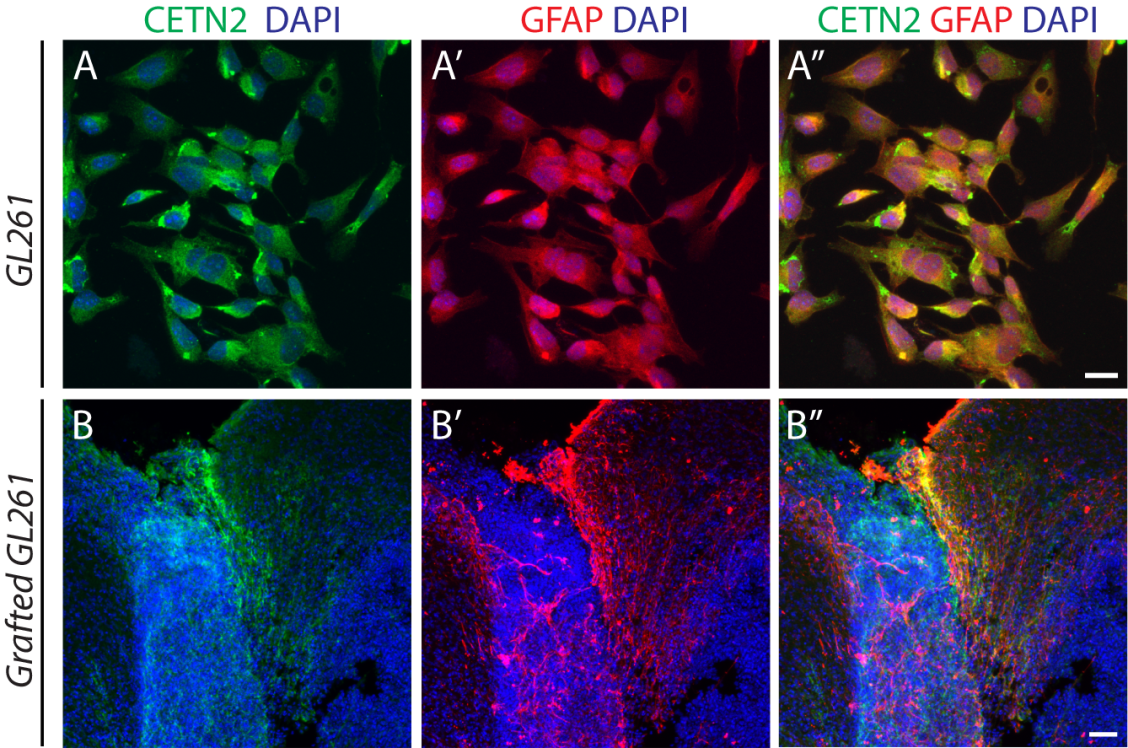


**Supplementary figure 6. CETN2 is expressed in mouse glioma cells. (A-A’’)** Cultured GL261 cells exhibit CETN2 and GFAP immunopositivity. (B-B’’) Upon implantation in mouse motor cortex GL261 cells retain GFAP immunopositivity and some CETN2 immunoreactivity. Scale bars: A-A’’ 20 µm; B-B’’ 50 µm.
